# Applying Principles of Implementation Science to Aging Programs and Policies

**DOI:** 10.1093/geroni/igab046.186

**Published:** 2021-12-17

**Authors:** Jaime Hughes, Nancy Morrow-Howell

## Abstract

Implementation science, defined by NIH as “the scientific study of the use of strategies to adopt and integrate evidence-based health interventions,” continues to grow within research, education, and practice-based settings. Building on principles from organizational psychology, intervention science, health economics, and health services research, implementation science aims to explore how, and under what conditions, evidence-based interventions are successfully implemented and sustained in real-world settings. Applying implementation science to aging programs and settings may help to accelerate the translation of effective programs and policies into practice. This interdisciplinary symposium will provide an introduction to key principles and applications of implementation science. The first three presentations will focus on largescale spread of interventions while the last two presentations will focus on broader applications of implementation science. The first two presentations will focus on adapting interventions from delivery in one setting or population to another. The third presentation will discuss the role of implementation strategies in scaling an intervention from a controlled research setting into a large integrated healthcare system. The third presentation will focus on the intersection of implementation science and policy. The final presentation will discuss the role of implementation science in alleviating health disparities and advancing health equity. Each presentation will utilize examples from ongoing research studies to demonstrate principles. The session will close with an interactive discussion on the role of implementation science within aging, including challenges and considerations for aging programs, policies, and populations as well as opportunities for further training and education.

